# A Novel Design Method of Gradient Porous Structure for Stabilized and Lightweight Mandibular Prosthesis

**DOI:** 10.3390/bioengineering9090424

**Published:** 2022-08-30

**Authors:** Renshun Liu, Yuxiong Su, Weifa Yang, Kai Wu, Ruxu Du, Yong Zhong

**Affiliations:** 1 Shien-Ming Wu School of Intelligent Engineering, South China University of Technology, Guangzhou 511400, China; 2 Oral and Maxillofacial Surgery, Prince Philip Dental Hospital, The University of Hong Kong, Hong Kong SAR 999077, China; 3 Guangzhou Janus Biotechnology Co., Ltd., Guangzhou 511400, China

**Keywords:** functionally graded design, mandibular prosthesis, mechanical properties, high porosity

## Abstract

Compared to conventional prostheses with homogenous structures, a stress-optimized functionally gradient prosthesis will better adapt to the host bone due to its mechanical and biological advantages. Therefore, this study aimed to investigate the damage resistance of four regular lattice scaffolds and proposed a new gradient algorithm for stabilized and lightweight mandibular prostheses. Scaffolds with four configurations (regular hexahedron, regular octahedron, rhombic dodecahedron, and body-centered cubic) having different porosities underwent finite element analysis to select an optimal unit cell. Meanwhile, a homogenization algorithm was used to control the maximum stress and increase the porosity of the scaffold by adjusting the strut diameters, thereby avoiding fatigue failure and material wastage. Additionally, the effectiveness of the algorithm was verified by compression tests. The results showed that the load transmission capacity of the scaffold was strongly correlated with both configuration and porosity. Scaffolds with regular hexahedron unit cells can withstand stronger loads at the same porosity. The optimized gradient scaffold showed higher porosity and lower maximum stress than the target stress value, and the compression tests also confirmed the simulation results. A mandibular prosthesis was established using a regular hexahedron unit cell, and the strut diameters were gradually changed according to the proposed algorithm and the simulation results. Compared with the initial homogeneous prosthesis, the optimized gradient prosthesis reduced the maximum stress by 24.48% and increased the porosity by 6.82%, providing a better solution for mandibular reconstruction.

## 1. Introduction

The mandible supports the contours of one-third of the lower face, maintains normal occlusion and vocal function, and plays a vital role in physical and mental health. Complex mandibular reconstruction is common due to a wide range of etiologies, including tumors, inflammatory diseases, fractures, or other factors [1]. Vascularized fibular flaps are considered the gold standard for mandibular reconstruction procedures [2]. However, the optimal reconstruction of mandibular defects to fully restore the aesthetic and functional aspects of the mandible remains challenging due to the complex anatomy of the head and neck [3]. Moreover, several studies have investigated complications related to the donor site, such as reduced walking endurance or strenuous exercise capacity [4,5].

Bone tissue engineering can offer a novel solution for repairing bone defects by integrating the merits of autografts and allografts while eliminating the mismatch between donor scarcity and key anatomic structures [6]. Due to its excellent biocompatibility, corrosion resistance, as well as good mechanical properties, Ti6Al4V alloy is best suited for orthopedic prosthesis production [7,8]. Since Young’s modulus for solid titanium prostheses is greater than that of the natural bone, it induces a stress shielding effect and compromises the bone-implant interface, leading to osteoporosis [9]. Ideally, the implants should have high porosity to reduce the stress shielding effects and allow the new bone to grow into the porous implant. Additionally, the highly porous implant should be strongly permeable to promote nutrient diffusion and deliver enough cellular mass for tissue repair [10,11]. Unfortunately, the scaffold porosity is negatively correlated with the bearing capacity, which is one of the limitations of the prosthesis for repairing bony defects.

In recent years, the development of the rapid prototyping technique has facilitated the fabrication of prostheses [12]. Compared with the conventional foam fabrication methods, such as metal deposition, infiltration casting, and powder metallurgy, the rapid prototyping technique enables a more accurate fabrication of porous structures by using digital models, which allows free designing of pore distribution regardless of manufacturing constraints [12].

Functionally gradient implants have attracted considerable attention because they combine mechanical and biological requirements to better simulate host tissues [13,14]. Based on the characteristics of design methods, the current functionally gradient implant design approaches are divided into three categories. For Type 1, the porous implant is designed according to the maximum stress capacity of the defect region. The Gibson and Ashby model states that the mechanical properties of porous structures are negatively correlated with the porosity, and the bearing capacity of the corresponding porosity can be obtained by fitting the porosity-elastic modulus curve with compression tests [15,16]. Xiao et al. designed a mandibular titanium-coated polymer lattice prosthesis in combination with the maximum stress distribution at the defect site, in which the lattice porosities corresponded with the maximum and minimum mandible stress regions, which showed a 20% gradient difference [17]. However, since the nodes in the periodic lattice structure did not fit properly on the designed surface, several free-ends and step surfaces were generated, leading to impaired clinical application. More importantly, optimization is still necessary in low-stress regions. In Type 2 design, the lattice struts are optimized by azimuth gradients according to the position of high- and low-stress regions along with the numerical simulation results. Small pore sizes or large strut diameters are used in high-stress regions to prevent fatigue failure, while large pore sizes or small diameters are used in low-stress regions to reduce material wastage [18,19,20]. Despite these measures, gradient optimization cannot be subdivided into each strut, and Boolean operations remain unavoidable. Lastly, the Type 3 gradient design displays accurate strut diameters for functional enhancement. Luo et al. [21] designed a tetrahedral structure implant by extracting mesh lines and following the stress homogenization principles for optimization. This resulted in increased porosity in the optimized implant when compared with the initial implant. However, only the tetrahedral unit cell structure can be designed; additionally, the maximum stress does not converge to the target stress value. All gradient implants are designed according to these three types. As far as the authors know, there are few design methods for functionally gradient structures that simultaneously cover the parameters of pore shape and size, maximum stress, and porosity.

Based on previous studies [21,22,23,24], four structures with the same lattice size and different porosities were simulated to obtain the optimal lattice unit cell with a stronger bearing capacity. A functionally gradient scaffold optimization algorithm was proposed to control maximum stress and improve porosity, that is, a larger diameter is designed for high-stress struts and a smaller diameter for low-stress struts to reduce material wastage as much as possible while maintaining their performance. Additionally, this functionally gradient optimization algorithm is applicable to a hexahedral mandibular prosthesis as well as other regular lattice implants.

## 2. Materials and Methods

### 2.1. Design of Porous Scaffolds

**Figure 1:**
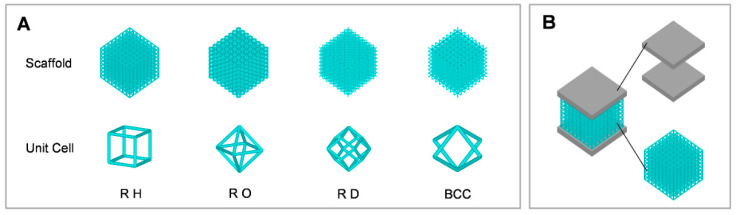
Design of porous scaffolds. (**A**) Configurations for four porous scaffolds. (**B**) Sample assembly of a porous scaffold (cyan) and compressed substrates (gray).

To detect the properties of a few lattices, four types of unit cells were chosen from previous research findings [25,26,27,28]: regular hexahedron unit cell (RH), regular octahedron unit cell (RO), rhombic dodecahedron unit cell (RD), and body-centered cubic unit cell (BCC) (Figure 1A). The three-dimensional (3D) scaffolds were established using regular, repeated lattice unit cells and modeled by Ansys 15.0 software (Dassault, Waltham, MA, USA). It was suggested that the pore sizes of 200–1200 µm are recommended for tissue generation [29]. Pore sizes were measured with the inner circle diameter in the pore shape [30]; a lattice size of 1.5 mm was regarded as the perfect size for promoting bone proliferation within the lower and upper limits. According to ISO 13,314 mechanical testing of metals, ductility testing, and compression tests should be conducted for porous and cellular metals. All spatial dimensions of the scaffolds should be at least 10 times the average pore size and not less than 10 mm, and the scaffold height-to-edge length ratio between 1 and 2. The cell size was set at 1.5 mm, and eight duplicated cells were kept in each of the three coordinate axes. The 3D scaffolds had a cube shape, displaying length, width, and height of 12 mm, 12 mm, and 12 mm, respectively. Based on this, three porosities were studied at 90% (P90), 80% (P80), and 70% (P70) to assess the role of porosity in the mechanical properties of the prosthesis.

### 2.2. Mechanical Evaluation of Porous Scaffolds

For evaluating the mechanical properties of porous scaffolds having varied unit cells and porosity, the scaffolds in each group were simulated by finite element analysis with Ansys software. Two plates (2 mm thick) were kept at the top and the bottom of the scaffold; all models had the same boundaries (Figure 1B). The lower surface of the composite model was fastened, and a vertical pressure (10 kN) was imposed on the upper surface. In this simulation model, the plate was the rigid body, while the scaffold material was Ti6Al4V alloy with Young’s modulus of 110 GPa and Poisson’s ratio of 0.3 [31]. The mesh sensitivity analysis reported accurate convergence of the mesh sizes of 0.15 mm and 0.5 mm for the scaffolds and the compressed substrates for all the simulations, respectively.

### 2.3. Optimization of Porous Scaffold

The numerical analysis results determined the scaffold with the strongest bearing capacity at the same porosity. An optimization algorithm was also developed for obtaining a lighter scaffold within the target stress value. In the scaffold, the struts fulfilled the minimum weight requirements of the target stress value, whereas the outer contour and scaffold elements were unchanged, with the addition of a strut diameter as a design parameter. The mathematical model for the optimization process, as well as achieving an expected target among all practical proposals, is expressed as:

(1) $$\mathrm{Find}\;D=[D_{1},D_{2},\ldots ,D_{n}],$$

(2) $$\mathrm{Min}\;W=\rho \sum_{i=1}^{n}l_{i}A_{i}=\frac{\rho \pi }{4}\sum_{i=1}^{n}l_{i}D_{{}_{i}}^{2},\;i=1,2,\ldots ,n,$$

(3) $$\sigma_{\max}{=\mathrm{Max}\text{\{}\sigma }_{i}\text{\}}<[\sigma ],\;i=1,2,\ldots ,n,$$

(4) $$s.t.\;D_{i}\in \left\{0.3,0.31,\ldots ,0.7\right\}\mathrm{mm},\;i=1,2,\ldots ,n,$$

where D represents the set of strut diameters of the scaffold, n refers to the total number of struts within the scaffold, $D_{i}$ means the diameter of the ith strut, W indicates the scaffold weight, ρ indicates the material density, $l_{i}$ and $A_{i}$ denote the length and cross-sectional area of the ith strut, respectively, $\sigma_{\max}$ signifies the maximum stress value within the scaffold, $\sigma_{i}$ stands for the maximum stress of the ith strut, and σ suggests the target stress value in iteration. Strut diameter falls within 0.3–0.7 mm (0.01 mm interval).

The pseudo-code algorithm is divided into several steps and is shown in Algorithm 1.
**Algorithm 1:** strut diameter optimization$\begin{array}{l} Input\text{:}\;\mathrm{iterations},\;k=0;\;\mathrm{strut}\;\mathrm{diameter},D^{\left(0\right)}=[D_{1}^{\left(0\right)},D_{2}^{\left(0\right)},\ldots ,D_{n}^{\left(0\right)}];\;\\ \;\mathrm{iteration}\;\mathrm{coefficient},\;\lambda^{{}_{\left(0\right)}}=1. \end{array}$$\begin{array}{l} Output\text{:}\mathrm{iterations},\;k;\;\mathrm{strut}\;\mathrm{diameter},\;D^{\left(k\right)}=[D_{1}^{\left(k\right)},D_{2}^{\left(k\right)},\ldots ,D_{n}^{\left(k\right)}];\;\\ \;\mathrm{peak}\;\mathrm{stress},\;\sigma^{\left(k\right)}. \end{array}$$\begin{array}{l} \;while\;\{\sigma^{\left(k\right)}\geq [\sigma ]\;and\;k\leq 100\}\|\;do \end{array}$$\;\bar{D_{i}^{\left(k+1\right)}}=\sqrt{\left(\lambda^{\left(k\right)}\sigma_{i}^{\left(0\right)}/\left[\sigma \right]\right)}\cdot D_{i}^{\left(0\right)},i=1,2,\ldots ,n$$\;if\;\bar{D_{i}^{\left(k+1\right)}}<0.3$$\;D_{i}^{\left(k+1\right)}=0.3$$\;else\;if\;\bar{D_{i}^{\left(k+1\right)}}\geq 0.7$$\;D_{i}^{\left(k+1\right)}=0.7$    else$\;\bar{D_{i}^{\left(k+1\right)}}\leq D_{i}^{\left(k+1\right)}<\bar{D_{i}^{\left(k+1\right)}}+0.01\;and\;D_{i}^{\left(k+1\right)}\in \{0.3,0.31,\ldots ,0.7\mathrm{mm}\}$   end if   Solve$\;\sigma_{i}^{\left(k\right)}=\max\left\{\sigma_{im}^{\left(k\right)}\right\},m\in \left\{1,2,\ldots ,q\right\}$$\;\sigma^{\left(k\right)}=\max\left\{\sigma_{i}^{\left(k\right)}\right\},i\in \left\{1,2,\ldots ,n\right\}$$\;\lambda^{{}_{\left(k+1\right)}}=1.1\lambda^{{}_{\left(k\right)}}$   k←k+1  end$\;return\;D^{\left(k\right)},\;\sigma^{\left(k\right)},\;\lambda^{\left(k\right)},k$

k means the number of iterations in Algorithm 1. $D^{\left(0\right)}$ represents the initial design, the superscript denotes the number of iterations, $D_{i}^{\left(0\right)}$ suggests the initial diameter of the ith strut, total n. $\lambda^{\left(k\right)}$ indicates the iteration coefficient of the kth iteration. ‘SOLVE’ represents the finite element solution of the model. $\sigma_{im}^{\left(k\right)}$ signifies the maximum stress of the mth loading during the kth iteration, m signifies the number of loading types with a total number of q. There is only one loading parameter for the compression experiment, i.e., q equals 1. $\sigma^{\left(k\right)}$ stands for the maximum stress value during the kth iteration of the scaffold.

In this study, the optimal configuration with 70% porosity was taken as the initial scaffold and was optimized with the target stress value to prevent scaffold failure and material wastage. The scaffold was also optimized with three target stresses to assess the effect of target stress on the results. The performance of the scaffolds was evaluated by the porosity level and maximum stress value, whereas the mechanical properties were verified by the compression test.

### 2.4. Test of Porous Scaffolds

Based on the virtual models, the initial and optimized porous scaffolds were prepared with a metal printer, Dimental-300 (Laseradd, Guangzhou, China). Finally, 24 3D porous Ti6Al4V scaffolds (six copies per group) were obtained. The SENS micro-electric universal test was used to conduct a uniaxial compression test until failure to evaluate the scaffold’s compression performance with a loading speed of 1 mm/min. The stress-strain curves and failure stress values were obtained by extracting the loading curve and calculating the load size at scaffold failure.

### 2.5. Prosthesis Design and Optimization for Mandibular Reconstruction

**Figure 2:**
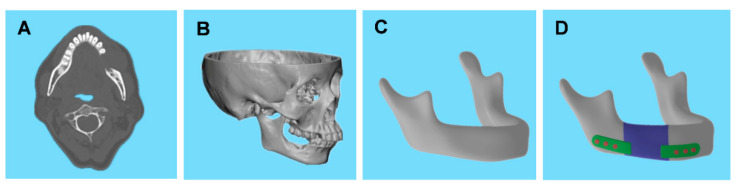
Mandible preparation and implant design. (**A**) CT image of defective mandible. (**B**) Three-dimensional model of maxilla and mandible. (**C**) Repaired mandible. (**D**) Reconstructed mandibular model, including residual mandible (gray), design model (blue), plates (green), and screws (orange).

The defective mandible was reconstructed with optimal configuration prosthesis in oral squamous cell carcinoma patients. Ethical approval and informed consent were obtained for using patients’ imaging data. Figure 2 shows the structural design preparation of mandibular defects. Mimics 20.0 (Materialise, Leuven, Belgium) was applied for contour extraction in maxillary and mandibular areas (Figure 2A) and obtaining the STL format model (Figure 2B). Furthermore, the mandibular coordinate was registered following facial feature points. Geomagic Studio 2012 (Geomagic, Morrisville, NC, USA) repaired the mandibular defect and checked the accuracy of the model to obtain the ideal mandibular model (Figure 2C). A design model was obtained according to the tumor resection plane and the directives of an experienced oral surgeon (Figure 2D). The plate was classified into two sections to facilitate stress transfer and decrease the stress shielding effects. The 8 mm wide plates were obtained by trimming the body 2 mm equidistant from the mandible surface, along with six cylindrical screws of radius 1.5 mm, with fastened plates at the residual mandible.

**Figure 3:**
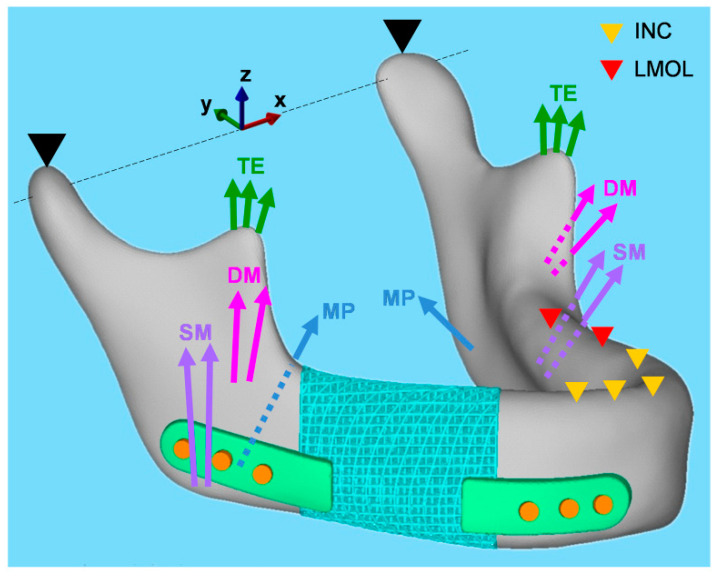
Muscular forces (arrow) and constraints (triangle) in the finite element model for simulation of the incisal clenching and left unilateral molar clenching tasks, respectively. SM, superficial masseter; DM, deep masseter; MP, medial pterygoid; TE, temporalis.

An optimal unit cell was used to construct the prosthesis and repair the mandibular defect. Finite element analysis of the prosthesis evaluated its mechanical properties under physiological loading conditions. Two static clenching tasks were simulated: incisal clenching (INC) and left unilateral molar clenching (LMOL) by applying muscle forces to the mandibular surface (Figure 3). The magnitude and direction of each muscle force were obtained from relevant studies [32,33], and Ti6Al4V alloy was used to manufacture the scaffolds, plates, and screws. The residual mandible was the cortical bone with an elastic modulus of 15 GPa and a Poisson’s ratio of 0.3 [34].

### 2.6. Statistics

The maximum von Mises stresses were calculated for each scaffold, along with a preliminary evaluation of data for accuracy. SPSS 24.0 software (IBM, Armonk, NY, USA) was used for one-way Analysis of Variance (ANOVA) of compressive strength, and the threshold value was defined as a *p*-value < 0.05. All data were generated using both instrumental measurements and rigorous evaluations.

## 3. Results

### 3.1. Mechanical Characterization of Porous Scaffolds

**Figure 4:**
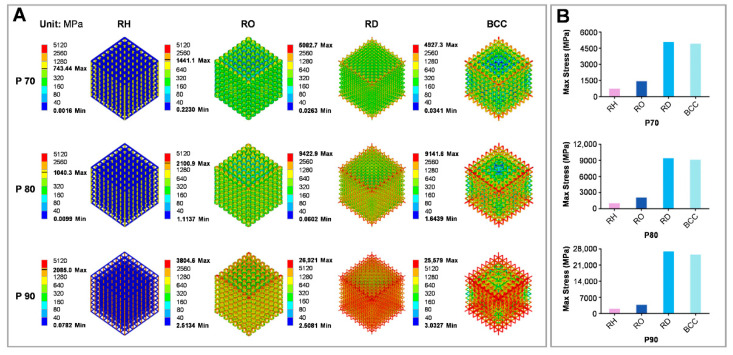
Porous scaffold’s mechanical properties assessed through finite element analysis. (**A**) Von Mises stress distribution for porous scaffolds with varying configurations and porosity under 10 kN compression. (**B**) Maximum von Mises stress values for porous scaffolds.

Figure 4A shows the von Mises stress distribution for every scaffold subjected to a vertical loading of 10 kN. Figure 4B shows the maximum stress value for porous scaffolds from 743 MPa (RH-P70) to 25,600 MPa (RD-P90); the maximum stress value of the RH unit was much lower than that of other scaffolds with the same porosity. A decrease in porosity led to a gradual reduction in maximum stress value for the same configuration, while the maximum stress for varying configurations having the same porosity was RD > BCC > RO > RH. Thus, RH exhibited superior performance to other groups of the same porosity.

### 3.2. Functionally Gradient Optimization of Porous Scaffold

The maximum stress value of an RH scaffold with 70% porosity was the minimum count under different configurations and porosities. Since the fatigue limit of rapid-prototyping Ti6Al4V porous structure decreases due to pores, cracks, and other factors, the actual fatigue limit is less than the theoretical value (900 MPa) and differs in various studies [35]. To prevent scaffold fatigue and verify the accuracy of the proposed algorithm, 700 MPa (Model B), 600 MPa (Model C), and 500 MPa (Model D) were taken as the target stresses, and the RH scaffold with 70% porosity was taken as the initial model (Model A) for optimization, respectively.

**Figure 5:**
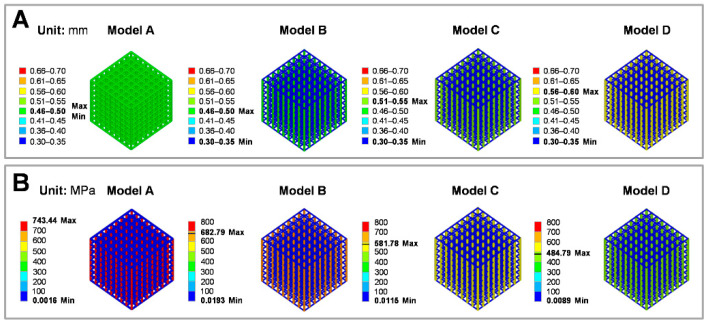
Geometric features and mechanical properties of the porous scaffolds. (**A**) Strut diameter distribution of the porous scaffolds, ranging from 0.3 mm to 0.7 mm (0.01 mm interval). (**B**) Stress distribution of porous scaffolds.

Figure 5A exhibits the diameter distribution for Model A–D. The outlines of the optimized scaffolds are similar to that of the initial scaffold. The strut diameters are represented by eight colors; warmer and colder colors denote larger and smaller diameters, respectively. In Model A, the whole scaffold displayed a relatively uniform distribution of strut diameters with 70% porosity. Although the initial design parameters were the same, the strut diameter in the direction of pressure increased gradually until 0.56 mm (Model D), followed by a decrease in the target stress value, and the strut diameters in other directions were at a minimum value. Figure 5B shows the stress distribution of Model A–D. The stress distribution of each optimized scaffold was similar to that of Model A; the stress of the strut in the direction of pressure was larger, while the strut in other directions displayed smaller stress. Consequently, the maximum stress of all optimized scaffolds converged to their corresponding target stress values. In Figure 5A,B, large strut diameters were designed for high-stress regions to reduce maximum stress, whereas small strut diameters were designed for low-stress regions to reduce material wastage. The scaffolds had different porosities and peak stress, as shown in Table 1, with 70% and 743.4 MPa for Model A, 86.5% and 682.8 MPa for Model B, 84.5% and 581.8 MPa for Model C, and 80.0% and 484.8 MPa for Model D, respectively.

**Figure 6:**
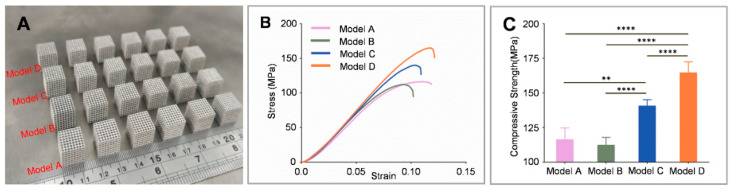
(**A**) As-built scaffolds. (**B**) Stress-strain curves of the compression tests of scaffolds. (**C**) Compressive strength of scaffolds (*n* = 6). Statistical analysis: one-way ANOVA (* *p* < 0.05; ** *p* < 0.01; *** *p* < 0.005; **** *p* < 0.001).

Figure 6A shows 24 additive manufacturing scaffolds (six copies per group). Figure 6B exhibits the stress-strain curves for scaffolds in compression tests. Subjected to low stress, the scaffolds demonstrated an elastic region with a high linearity level, and as the stress level increased to the critical value, the stress-strain curve increased slowly until it reached the crest and destroyed a part of the lattice. According to the stress-strain curves, the loading-bearing capacity of the scaffolds was similar to the simulation results and increased with a decrease in the target stress value. The reduced target stress value led to a gradual increase in the compressive strength that was displayed as: Model B < Model A < Model C < Model D. The maximum force for porous scaffolds was from 16,153 ± 974 N (Model B) to 23,684 ± 1091 N (Model D), thus, Model D displayed superior performance to other groups (Figure 6C). The apparent trend for stress-strain curves and compressive strength in different scaffolds was in agreement with the simulation analysis results.

### 3.3. Functionally Gradient Porous Mandibular Prosthesis

The mandibular prosthesis was constructed with an RH lattice structure with an initial strut diameter of 0.5 mm and 68.49% porosity. The strut diameter of the optimized prosthesis was 0.3–0.7 mm (0.01 mm interval), and the target stress value was 200 MPa. The mesh sensitivity analysis demonstrated accurate convergence with a mesh size of 0.15 mm for the porous prosthesis and 0.6 mm for other models for all simulations (139,058 nodes and 454,329 elements).

**Figure 7:**
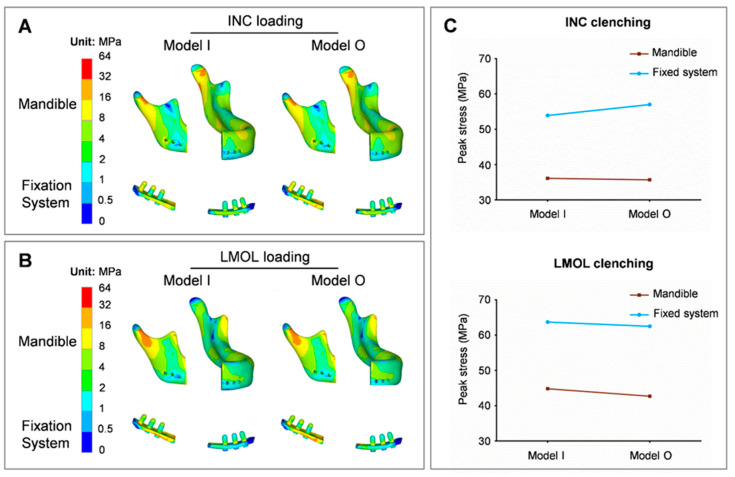
Von Mises stress distribution for mandible and fixation system subjected to INC loading (**A**) and LMOL loading (**B**), and the line chart for their maximum stress values (**C**).

The stress nephogram of the residual mandible and fixation system in the initial prosthesis (model I) and the optimized prosthesis (model O) installation, depicting the effect of the optimized prosthesis on the mandible and fixation system, is shown in Figure 7. The gradient optimization of the prosthesis under two clenching tasks, INC and LMOL, had no significant effect on the location of the high-stress region of the mandible and fixation system (Figure 7A,B). The maximum stress of the mandible was primarily concentrated in the condylar neck region, and the maximum stress of the fixation system was concentrated in the region surrounding the porous prosthesis. Prosthesis optimization had no significant effect on the peak stress values of the mandible and fixation system (Figure 7C).

**Figure 8:**
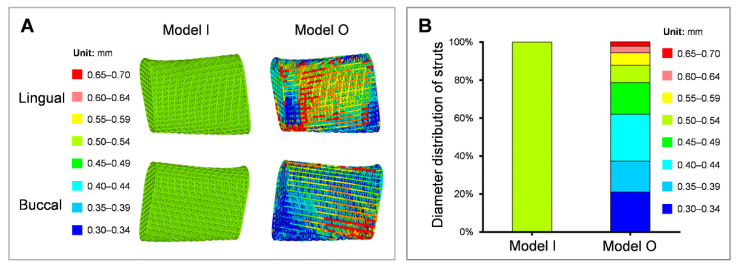
Geometric features (**A**) and strut diameter distribution (**B**) of the porous prostheses.

Figure 8A shows the geometric features of the prosthesis. The prosthetic strut diameters are marked in eight colors, in which warmer and colder colors indicate larger and smaller diameters, respectively. Model I displayed a uniform mesh in the whole porous prosthesis with a strut diameter of 0.5 mm and 68.49% porosity. The struts with large diameters were mainly concentrated at the prosthesis-plates connection regions and the lower part of the prosthesis closer to the mandibular ramus. The majority of the strut diameters of Model O decreased, while a few strut diameters increased when compared with Model I (Figure 8B).

Table 2 exhibits the porosity and maximum stress distribution of the prosthesis. The maximum stress of the initial prosthesis was 228.33 MPa and 261.35 MPa subjected to INC and LMOL loading, respectively. The maximum stress of the optimized gradient prosthesis was less than the target stress (200 MPa) and displayed increased porosity, thus, achieving the required efficacy.

**Figure 9:**
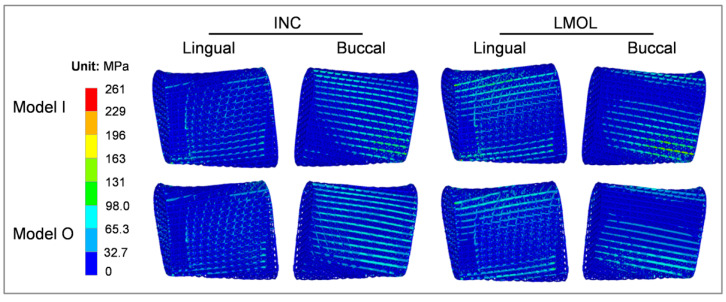
Von Mises stress in Model I and Model O under INC and LMOL loading.

Figure 9 exhibits the stress distribution for the initial and optimized prostheses. They shared the same stress distribution and were subjected to the same load. The maximum stress was distributed at the lower part of the prosthesis closer to the mandibular ramus in the LMOL loading task, while the high-stress struts were primarily concentrated on the prosthesis surfaces. Based on von Mises stress distribution in the initial homogenous prosthesis (Figure 9) and the diameter distribution of the optimization gradient prosthesis (Figure 8), the stress magnitude distribution regions were in accordance with the strut diameter size, as larger strut diameters reduced stress in high-stress regions, but smaller strut diameters reduced the material wastage of low-stress regions. It also increased the porosity and reinforced the prosthesis stability.

**Figure 10:**
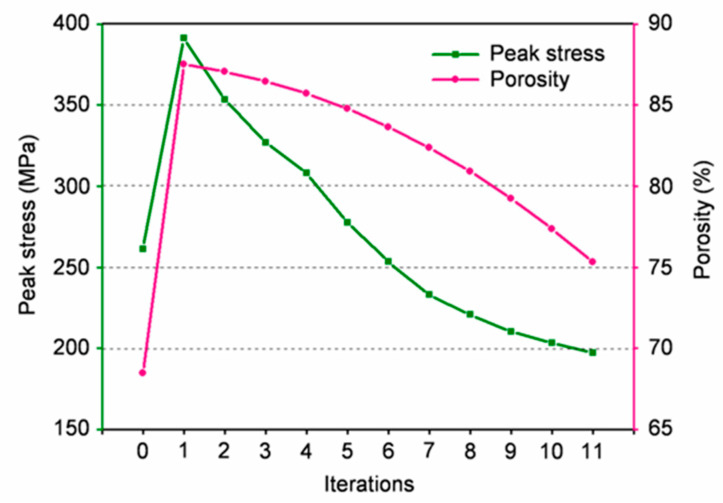
Variation of peak stress and porosity during the iteration process.

Figure 10 shows the change in the maximum stress value and prosthesis porosity in the optimization process. The first iteration increased the value of the maximum stress and porosity. As it continued, the maximum stress and porosity further decreased until the maximum stress was less than the target stress (200 MPa) in the eleventh iteration, leading to the end of the iteration. Eventually, the gradient prosthesis showed a 24.48% reduction in maximum stress, concurrent with a 6.82% increase in porosity when compared with the initial homogenous prosthesis, and proved the favorable performance of the gradient prosthesis.

**Figure 11:**
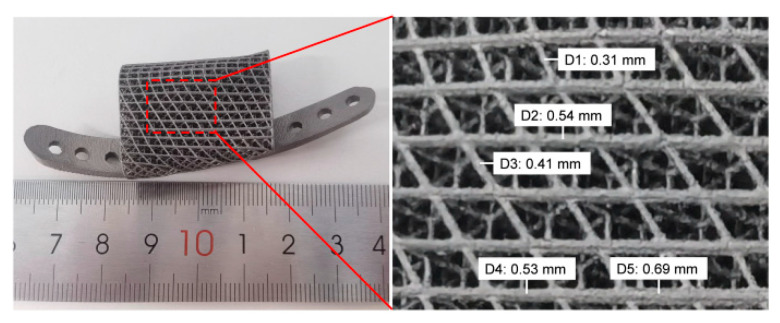
The printing details of the optimized prosthesis and the theoretical diameter (D1–D5) of part struts.

Ti6Al4V alloy was used to prepare the initial and optimized prosthesis (including plates) by a metal printer. The masses of the initial and optimized prostheses calculated with the Sartorius high-precision electron balance were 11.9 g and 10.5 g, respectively. Figure 11 shows the details of the optimized prosthesis. The strut of the gradient prosthesis was fully printed and presented a gradient distribution, and the connection was complete and closely connected, implying that the diameter sizes for the prosthesis chosen for the current study were rational and were well suited for manufacturing.

## 4. Discussion

An excellent prosthesis used for repairing large segmental bone defects can duly recover the physiological function and facilitate bone regeneration at the defect site. An ideal bone prosthesis should be biomechanically stable, able to match the mechanical properties of the defective bone to avoid implant failure or stress shielding, and possess high porosity and interconnection that allows cell migration, proliferation, and differentiation, as well as nutrient-waste exchange [11]. Compared with the uniform regular lattice structure, the functionally gradient prosthesis can better simulate the mechanical properties of human bone and enhance the implant’s success by displaying sufficient porosity.

It has been well established that the bearing capacity of different scaffolds is discrepant among various configurations [28]. To obtain a high-strength scaffold with high porosity, four scaffolds with different configurations and porosity were selected and underwent finite element analysis to evaluate their mechanical properties under the same conditions (Figure 4). The maximum von Mises stress of the scaffolds gradually reduced as the porosity decreased in all the groups. This finding was consistent with the outcome of the Gibson–Ashby model, which stated that porosity is negatively correlated with the bearing capacity of the porous structure. Meanwhile, the configuration also affected the scaffolding failure and was consistent with relevant literature [22]. Subjected to identical loading conditions, the maximum stress value of the RH scaffold at 70% porosity was the lowest and below the fatigue limit. Since the RH scaffold has struts along the direction of the pressure, it might have improved the scaffold’s bearing capacity; changing the loading direction may result in slight fluctuations due to the absence of isotropy.

A gradient optimization algorithm was proposed for obtaining a scaffold with controllable maximum stress and high porosity. Taking the RH scaffold with 70% porosity as an example, most of the struts were at low-stress levels, and parts were at high-stress levels, indicating that most of the struts were underutilized and a few were overutilized. The strut diameter of the scaffold could be adjusted within the design range to realize a stabilized and lightweight scaffold. For accurate usage of selective laser melting technology, the lower limit of the strut diameter was set to 0.3 mm to guarantee optimum scaffold forming quality, whereas the upper limit of the scaffold diameter was determined by the load. The maximum stress was found to be 321.06 MPa with a strut diameter of 0.7 mm, which was smaller than the target stress value and assured the convergence of iteration. The upper limit of the strut diameter had a positive correlation with the convergence speed; smaller values might cause non-convergence, while larger values could cause shrinkage or closure of partial pores.

According to our results, the maximum stresses in optimized scaffolds were lower than the target stress value; all optimized scaffolds displayed increased porosity and showed a negative correlation with the target stress value. The reason was that in order to obtain the scaffolds with low maximum stress, the diameters of struts with high stress were increased; to reduce material waste, the diameters of struts with low stress were decreased. A lower target stress value required more struts to increase the diameter, resulting in a decrease in the scaffold’s porosity. There is a gradient relationship between the diameter distribution of the scaffold and stress change. The gradient scaffold had higher porosity and a lower maximum stress value to enhance permeability and prevent scaffold damage as compared to the initial homogenous scaffold, which verified the effectiveness and feasibility of the optimization algorithm.

Furthermore, compression tests were conducted on scaffolds to enhance the accuracy of the finite element analysis results. The stress-strain curves were consistent with the finite element analysis results, while the elastic modulus and compressive capacity of the gradient scaffolds increased as the target stress value decreased. The compressive strength of model B was slightly lower than model A. The reason was that the smaller maximum stress values of the strut in the vertical direction of pressure caused a reduction in the corresponding strut diameter; however, due to the deviation of manufacturing and test conditions, the struts in the vertical direction of pressure resisted a part of the vertical pressure, leading to minor changes in the compressive strength.

There has been extensive research to determine the range of fatigue properties of additive manufacturing (AM) products. Due to the influence of residual stresses, pores, and other factors, the actual yield stress of Ti6Al4V alloy is far less than the theoretical limit value (900 MPa) [36]. The fatigue strength for Ti6Al4V porous, cellular structures printed by using AM products, possesses 20–30% of the theoretical yield strength [37]. According to Nikolas’s study [38], the fatigue limit stresses of as-built and stress-relieved Ti6Al4V samples manufactured using electron beam melting (EBM) were 200–250 MPa at 10 million cyclic stresses. Therefore, choosing 200 MPa as the limit stress value proved more beneficial for maintaining the stability of the prosthesis.

Based on our study, a personalized mandibular prosthesis with an RH unit cell was constructed and optimized according to the proposed algorithm. Compared to the initial homogeneous prosthesis, the maximum stress for gradient prosthesis was smaller than the target stress value (200 MPa), with a 24.48% decrease and a 6.83% increase in maximum stress and porosity, respectively. Since mandibular occlusion is complex, several mastication load forces are simultaneously considered, which makes the implementation of the traditional optimization methods difficult. The optimization algorithm proposed in our study can record the stress of the struts under several loadings simultaneously and use the maximum stress distribution to carry out iterative optimizations with stronger adaptability. The design and optimization approach developed by the current research applies to gradient lattice mandibular prosthesis and provides a reference for the stabilized and lightweight design of other orthopedic implants. The prosthesis design method proposed in this study can also be applied to other materials (such as TiZr or ceramic materials).

The limitations of our study are as follows. Firstly, we assumed that the bone replacement material was homogeneous and isotropic, which was difficult to achieve due to the limited processing conditions. Secondly, we only explored the mechanical properties of four common configurations of porous scaffolds, and more configurations need to be considered to fabricate an optimal scaffold. Thirdly, the mandibular tooth distribution affects the prosthesis stress distribution. The gradient optimization algorithm proposed in this study is also suitable for the case that considers teeth, and we will retain the dentition in future studies. Finally, to our knowledge, there were few reports on muscle strength after mandibular reconstruction, and it was unclear whether reconstruction had any effect on muscle forces. We only considered the ideal condition in which there was no change in muscle strength before and after mandibular reconstruction.

## 5. Conclusions

This study proved the accuracy of different configurations and porosity in modifying the inherent mechanical properties of porous scaffolds. An optimization algorithm was proposed to modify the regular hexahedral scaffold with a porosity of 70% and showed that the optimized gradient scaffolds have higher porosities and lower maximum stresses than the target stress values. Furthermore, compression tests also verified the algorithm’s authenticity. A regular hexahedral mandibular prosthesis was constructed and optimized with the help of the algorithm that displayed a gradient change in the strut diameter, with a larger diameter in the high-stress regions (and vice versa). Consequently, there was a reduction in maximum stress by 24.48% to avoid fatigue failure and an increase in porosity of 6.83% to reduce material wastage. However, designing a mandibular prosthesis mimicking the performance of the host bone in terms of morphology and compression properties is a challenge that has long bothered researchers and requires more investigations on graded scaffolds in the future. We hope that the gradient prosthesis design algorithm presented in this study will provide valuable insights into the development of three-dimensional printed orthopedic implants.

## Figures and Tables

**Table 1:** The performance parameters for porous scaffolds, covering porosity and peak stress.

-	Target Stress (MPa)	Porosity (%)	Peak Stress (MPa)
Model A	-	70.00	743.44
Model B	700	81.86	682.79
Model C	600	79.63	581.78
Model D	500	77.68	484.79

**Table 2:** The performance parameters for prosthesis, covering porosity and peak stress under two clenching loadings.

-	Porosity [%]	Peak Stress [MPa]	
		INC	LMOL
Model I	68.49	228.33	261.35
Model O	75.32	151.20	197.37

## Data Availability

Not applicable.
